# Dietary Diversity in Cambodian Garment Workers: The Role of Free Lunch Provision

**DOI:** 10.3390/nu10081010

**Published:** 2018-08-03

**Authors:** Jan Makurat, Eleonore C. Kretz, Frank T. Wieringa, Chhoun Chamnan, Michael B. Krawinkel

**Affiliations:** 1 Institute of Nutritional Sciences, Justus Liebig University Giessen, Wilhelmstrasse 20, 35392 Giessen, Germany; eleonore.kretz@ernaehrung.uni-giessen.de (E.C.K.); krawinkel@fb09.uni-giessen.de (M.B.K.); 2 UMR 204 Nutripass, Institut de Recherche pour le Développement (IRD), IRD/UM/SupAgro, 911 Avenue d’ Agropolis, 34394 Montpellier, France; franck.wieringa@ird.fr; 3 Department of Fisheries Post-Harvest Technologies and Quality Control (DFPTQ), Fisheries Administration, Ministry of Agriculture, Forestry and Fisheries (MAFF), 186 Preah Norodom Boulevard, 12000 Phnom Penh, Cambodia; chhounchamnan@gmail.com

**Keywords:** dietary diversity, lunch provision, staff canteen, garment factory, Cambodia, industrial worker

## Abstract

The objective of this paper is to compare food consumption by Cambodian garment workers with and without access to a free model lunch provision through a factory-based canteen. Data from an exploratory randomised controlled trial were analysed. In total, 223 female Cambodian garment workers were allocated to an intervention arm (six-month lunch provision) or a control arm. Dietary intake on workdays was assessed by qualitative 24-h recalls at baseline and twice at follow-ups during the period of lunch provision using the Food and Agricultural Organization (FAO) guideline on assessing women’s dietary diversity. In total, 158 participants provided complete data on the dietary intake over workdays at all interviews. Lunch provision resulted in a more frequent consumption of dark green leafy vegetables (DGLV), vitamin A-rich fruits, other fruits, and oils and fats during lunch breaks. In contrast, flesh meats, legumes, nuts and seeds, as well as sweets, were eaten at a lower frequency. Except for a higher consumption rate of vitamin A-rich fruits and a lower intake frequency of sweets, lunch provision had a less clear impact on total 24-h intake from different food groups and was not associated with a higher women’s dietary diversity score (WDDS). A more gap-oriented design of the lunch sets taking into account underutilised foods and the nutritional status of the workers is recommended.

## 1. Introduction

The setup of staff canteens serving free lunch in Cambodian garment factories has been proposed as a suitable intervention to improve the dietary intake and the nutritional and health status of socially disadvantaged employees [1]. However, there is a lack of insight concerning the true consequences of lunch provision. The vast majority of factories does not have a canteen, with operation costs being the most critical factor [1]. The Lunch Provision in Garment Factories (LUPROGAR) study was a factory-based exploratory randomised controlled trial to assess the effects of a six-month low-price model lunch provision through a canteen during workdays on the nutritional status (anthropometry and micronutrient status) of female garment workers in Cambodia. Prior to this paper, the authors provided detailed information on the participants’ nutritional and health status at baseline [2], as well as on the low-price model lunch provision approach within the trial [3]. The objective of the present survey is to compare the frequency of consumption of food groups (at lunch and in total over 24 h) between study subjects with and without access to the model lunch provision.

## 2. Materials and Methods 

### 2.1. Study Setting

The LUPROGAR trial was implemented in 2015 at Apsara Garment Co. Ltd., an export-oriented garment factory located in the suburban commune Chom Chau of Phnom Penh, the capital of Cambodia. The majority of the 1300 employees were young unmarried women from low-income rural households. Conditions of employment were assumed to be comparable with overall working conditions in the sector. Apsara Garment Co. Ltd. operated on six workdays per week and was selected purposely since the management showed interest in collaborating in this research.

### 2.2. Participants

The study population included young non-pregnant nulliparous females employed by Apsara Garment Co. Ltd. The recruitment procedure has been described previously in detail [2]. In brief, signed informed consent forms were obtained from interested workers at lunch breaks and after work (middle of March until the beginning of April 2015), prior to any data collection. Workers who signed the informed consent were invited to the enrolment and baseline assessment (end of April 2015), which included a clinical screening. Background information on baseline sociodemographic characteristics, anthropometry, and haemoglobin and micronutrient status of the enrolled subjects can be found elsewhere [2].

### 2.3. Randomisation

Enrolled participants were individually allocated in equal shares into an intervention arm (access to six-month free lunch provision through canteen during workdays) and a control arm (equal monetary compensation at the end of the trial). A random variable (a/b) was assigned to each registered subject by making use of the random number generator within SPSS (v.22, IBM Corp., Armonk, NY, USA).

### 2.4. Lunch Provision

A temporary canteen was installed in a roofed outdoor area at the factory site specifically for this trial [3]. Adequate full lunch sets (consisting of a stir-fried dish, a soup, a side item (cooked rice), and a fruit dessert) were provided in collaboration with Hagar Catering and Facilities Management Ltd., an established Phnom Penh-based canteen service provider. Sets should provide about one-third of the recommended dietary allowance (RDA) for non-pregnant women aged 19–30 years old (total roughly 700 kcal) [4]. Based on these standards, a biweekly menu (including 12 model lunch sets) was outlined in consultation with the caterer [3]. Focus was laid on acceptable Cambodian dishes, using local foods and ensuring variety by providing cereals, various vegetables, animal source foods (meat or fish), and fresh fruits on a daily basis. Lunch provision for the intervention group was carried out by the caterer for six months from beginning of May till end of October 2015. Access to the canteen was voluntary and recorded daily. Additional information on exact costs, components and ingredients, serving sizes, and corresponding nutritive value of single lunch sets can be found elsewhere [3].

### 2.5. Data Collection

Dietary intake on workdays was assessed at baseline and at two follow-up interviews during the six-month lunch provision period (first follow-up at 2.5 months and second follow-up at 5 months) using the Food and Agricultural Organization (FAO) guideline and questionnaire on recording individual dietary diversity [5]. Subjects were asked to recall all foods and drinks they had consumed in the previous 24 h (always a workday). In the case of composite dishes, respondents were asked in detail for each individual ingredient, following a list of ingredients that was generated beforehand (including individual ingredients in lunch sets served at the canteen). All foods and drinks mentioned were then categorised into 16 food groups [5]. The food groups covered were noted separately for breakfast, lunch, and dinner, as well as for total 24-h intake. Skipped meals were recorded. Additional questions on home-prepared foods and purchasing at lunch breaks were added to the initial FAO questionnaire. The women’s dietary diversity score (WDDS) was calculated with data on total 24-h intake [5].

### 2.6. Statistical Analysis

The sample size calculation is described in detail in a previous paper [2]. Data from the questionnaires were double-entered using EpiData (v.3.1, EpiData Association, Odense, Denmark) while data management and analyses were executed using SPSS (v.22, IBM Corp.). The evaluation in this survey only included participants with complete data on workday dietary intake at all interviews. Differences between groups were tested using Fisher’s exact test in nominal variables and independent Student’s *t*-test in the continuous variable WDDS. Inequalities were considered statistically significant at *p* < 0.05.

### 2.7. Ethical Approval

The trial was conducted according to the guidelines laid down in the Declaration of Helsinki. Approval was obtained from the Institutional Review Board of the Faculty of Medicine at Justus Liebig University, Giessen, Germany (Identifier: 198/14) and the National Ethics Committee for Health Research at the Ministry of Health, Phnom Penh, Cambodia (Identifier: 0363 NECHR). Written informed consent was collected from all participants. The trial was registered at the German Clinical Trials Register (Identifier: DRKS00007666).

## 3. Results and Discussion

From a total of 267 workers who signed the informed consent, 229 were present at enrolment and 223 were randomly assigned to the control (*n* = 112) and the intervention group (*n* = 111) [2]. Baseline sociodemographic data are presented elsewhere [2] and equivalence amongst the groups was given. In total, 172 participants (*n* = 86 in each arm) completed the overall trial. All dropouts occurred equally distributed in both groups within the first two months. Unexpectedly, a part of the total factory staff, and therefore also women who already signed consents or were enrolled, ceased to work and left the factory during the initial study period. Daily lunch provision had great acceptance among the intervention subjects, who on average visited the canteen on 85% of the intervention days. In total, 158 subjects (*n* = 80 control and *n* = 78 intervention) had complete data on dietary intake for the workdays at all interviews (14 subjects did not work at the factory on the previous day at one or more of the assessments). 

Table 1 presents details about the dietary intake at lunch breaks by group. Before the study, 14% of participants consumed some types/items of home-prepared foods, whereas the vast majority (96%) purchased food and drinks in front of the factory gates. Foods most commonly consumed at lunch were cereals (100%, solely rice), flesh meats (72%), other vegetables (60%), sweets (54%), oils and fats (53%), fish and seafood (49%), dark green leafy vegetables (DGLV) (47%), and other fruits (44%). The frequency of consumption of vitamin A-rich fruits (1%), milk and milk products (1%), organ meat (2%), white roots and tubers (4%), and eggs (7%), was low. On average, subjects’ lunch meals at baseline were composed of 6.5 (standard deviation (SD) = 1.8) food groups and represented the most diverse meal as compared to breakfast and dinner (food group intake at breakfast/dinner not shown). There were no baseline differences between groups in variables of dietary intake at lunch breaks. 

The frequency of workers with home-prepared food for lunch breaks in the intervention group expectedly dropped to 0% at follow-ups. Since access to free lunch provision resulted in saving time and effort on food preparation it might also decrease the risk of lack of food safety as lunch boxes are usually stored without cooling on factory grounds [1]. Significantly fewer intervention participants reported purchasing food/drinks at follow-up interviews. Still, the proportion remained surprisingly high, although the lunch provision also included unlimited access to drinking water at the canteen. Intervention subjects mostly reported additional purchase of beverages and sweets after having visited the canteen.

At both dietary re-assessments, the lunch intake by intervention subjects was significantly higher for DGLV, vitamin A-rich fruits, other fruits, and oils and fats. On the other hand, access to lunch provision was also significantly associated with a lower rate in consumption of flesh meats, legumes, nuts and seeds, sweets (all at both follow-ups), and eggs (only at 5 months). 

The served lunch sets included various vegetables (often DGLV), fruits (including vitamin A-rich fruits), as well as a small amount of cooking oil [3]. The lower consumption rate of flesh meats at lunch can be attributed to the regular serving of fish portions in model lunch sets [3]. Only a few dishes served at the canteen included legumes and none contained nuts. Eggs were only served once within the biweekly model menu [3]. Control participants often reported lunch intake of soy bean products and/or groundnuts, as total participants did at baseline. As sweets were not provided at the canteen, any consumption of sweets in intervention women rested on additional purchase outside the factory. Nevertheless, a lower intake of free sugar among workers is regarded as beneficial [6]. Given the overall low rate in organ meats consumption at lunch breaks and the low total iron content in the lunch sets [3], future concepts should incorporate more of these haem-iron rich foods [7].

**Figure 1:**
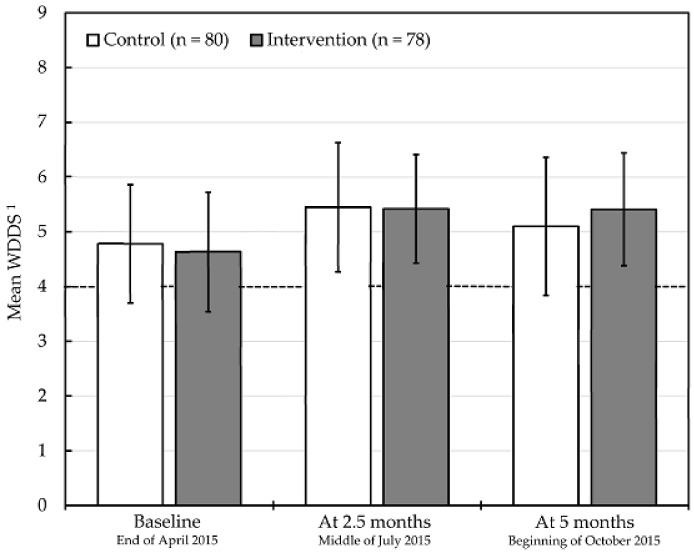
Mean women’s dietary diversity score (WDDS) at baseline and follow-up interviews in female Cambodian garment workers without (control) and with access to the six-month free model lunch provision through a canteen (intervention). Evaluation in participants with complete data on workday dietary intake at all interviews. Lines within bars illustrate the standard deviations. The dashed line indicates a cut-off for “inadequate” (WDDS < 4) and “adequate” (WDDS ≥ 4) dietary diversity [8]. Group comparisons using Student’s independent *t*-test showed no significant differences. ^1^ Aggregated continuous indicator (0–9), based on total 24-h consumption of starchy staples (cereals and/or white roots and tubers); dark green leafy vegetables; vitamin A-rich fruits and vegetables (vitamin A-rich vegetables and tubers and/or vitamin A-rich fruits); other fruits and vegetables (other fruits and/or other vegetables); organ meat; meat and fish (flesh meats and/or fish and seafood); eggs; legumes, nuts and seeds; and milk and milk products [5].

Table 2 and Figure 1 present the data on total 24-h dietary intake and dietary diversity scores during workdays by group. Initially, there were no differences between the groups. At baseline, 10% of subjects skipped breakfast. Skipping of breakfast in intervention participants increased to 21% at 2.5 months, however, no significant difference in skipping breakfast between groups was observed at both follow-up interviews. Skipping of meals in garment workers with access to a staff canteen should be closely monitored, as it might counteract the expected benefits from lunch provision. With increasing rates of women skipping breakfast, lunch programmes for workers might need to consider additional meal/snack provision in the morning. 

Overall, baseline 24-h intake from the different food groups was characterised by consumption of cereals (100%, mainly rice), oils and fats (89%), flesh meats (89%), other vegetables (80%), fish and seafood (75%), DGLV (74%), sweets (67%), other fruits (55%), vitamin A-rich vegetables and tubers (42%), and legumes, nuts and seeds (34%). The mean WDDS at baseline was 4.7 (SD = 1.1), representing an “adequate” dietary diversity on average [8]. Although the consumption of iron-rich foods (flesh meats and fish) was common, the prevalence of low iron status at baseline was high in enrolled subjects [2]. The quantities of these foods might have not been sufficient to meet the RDA for iron as reported for Cambodian women in rural areas [9].

The evaluation of the 24-h food group intake at follow-up interviews showed a differentiated impact of lunch provision. On the one hand, the intake of vitamin A-rich fruits was significantly higher and the consumption of sweets was significantly lower in intervention subjects at both re-assessments. On the other hand, a significantly higher rate in intake of other fruits, vitamin A-rich vegetables and tubers, and DGLV, as well as a significantly lower intake of legumes, nuts and seeds, and eggs, was noted only for one of the two follow-up interviews. Different to the intake from food groups during lunch breaks, no differences were observed between the intervention and the control group for the 24-h dietary intake of flesh meats. 

Although the mean WDDS among the intervention group increased from 4.6 (SD = 1.1) at baseline to 5.4 (SD = 1.0) at both follow-ups, no significant differences in WDDS between groups could be observed, which is assumed to be in line with the lunch provisions’ overall little impact on 24-h food group intake. The mean WDDS in control subjects was 5.4 (SD = 1.2) and 5.1 (SD = 1.3) at 2.5 and 5 months, respectively.

### Limitations of the Study

The trial’s model lunch provision was not specifically designed for improving the intake of specific foods nor the overall dietary diversity. Moreover, the enrolled women generally showed a relatively diverse total dietary intake, given their mean WDDS of 4.7 at baseline. Furthermore, as the calculation of the sample size for the LUPROGAR trial was based on different outcomes [2], it is not fully appropriate for the evaluation of frequencies of food group consumption. Given the relatively small sample size within this trial, inequalities between the control and the intervention group had to be marked to reach statistical significance. At last, no correction for multiple comparisons was conducted, which is in line with recommendations for exploratory studies [10].

## 4. Conclusions

LUPROGAR’s low-price model lunch provision for Cambodian garment workers resulted in a more frequent consumption of DGLV, vitamin A-rich fruits, other fruits, and oils and fats during lunch. In contrast, it was likewise associated with a lower intake frequency of flesh meats, legumes, nuts and seeds, and sweets. Future model lunch sets for this group of women should incorporate some organ meats to increase the provision of iron. Beside a higher consumption rate of vitamin A-rich fruits and a lower intake frequency of sweets, lunch provision had a less clear impact on total 24-h intake from different food groups and was not associated with a higher WDDS. A more gap-oriented design of the lunch sets taking into account underutilised foods and the nutritional status of the workers is recommended for increasing their WDDS. Finally, skipping of meals in workers with access to a staff canteen should be closely monitored in order to avoid unfavourable dietary changes.

## Figures and Tables

**Table 1:** Home-prepared food, purchasing, and food group intake at lunch breaks at baseline and follow-up interviews in female Cambodian garment workers without (control) and with access to the free model lunch provision through a canteen (intervention). ^1^

	Baseline (End of April 2015)			At 2.5 Months (Middle of July 2015)			At 5 Months (Beginning of October 2015)		
	Control (*n* = 80)	Intervention (*n* = 78)		Control (*n* = 80)	Intervention (*n* = 78) ^2^		Control (*n* = 80)	Intervention (*n* = 78) ^3^	
Variables	*n* (%)	*n* (%)	*p*	*n* (%)	*n* (%)	*p*	*n* (%)	*n* (%)	*p*
Home-prepared food for lunch break	11 (14)	11 (14)	ns	15 (19)	0 (0)	<0.001	5 (6)	0 (0)	0.059
Purchased food/drinks at lunch break	76 (95)	76 (97)	ns	77 (96)	43 (55)	<0.001	77 (96)	46 (59)	<0.001
Food group intake at lunch break									
Cereals	80 (100)	78 (100)	-	80 (100)	78 (100)	-	80 (100)	78 (100)	-
Spices, condiments, and beverages	79 (99)	77 (99)	ns	79 (99)	78 (100)	ns	79 (99)	78 (100)	ns
Flesh meats	58 (73)	55 (71)	ns	57 (71)	39 (50)	0.009	68 (85)	43 (55)	<0.001
Other vegetables	52 (65)	43 (55)	ns	52 (65)	54 (69)	ns	56 (70)	59 (76)	ns
Sweets	44 (55)	42 (54)	ns	57 (71)	27 (35)	<0.001	54 (68)	29 (37)	<0.001
Oils and fats	47 (59)	36 (46)	ns	52 (65)	73 (94)	<0.001	58 (73)	74 (95)	<0.001
Fish and seafood	43 (54)	35 (45)	ns	36 (45)	42 (54)	ns	37 (46)	36 (46)	ns
Dark green leafy vegetables	36 (45)	38 (49)	ns	41 (51)	62 (80)	<0.001	38 (48)	58 (74)	0.001
Other fruits	37 (46)	33 (42)	ns	49 (61)	70 (90)	<0.001	48 (60)	62 (80)	0.009
Legumes, nuts and seeds	22 (28)	23 (30)	ns	35 (44)	13 (17)	<0.001	29 (36)	16 (21)	0.035
Vitamin A-rich vegetables and tubers	19 (24)	22 (28)	ns	27 (34)	31 (40)	ns	28 (35)	35 (45)	ns
Eggs	8 (10)	3 (4)	ns	11 (14)	13 (17)	ns	13 (16)	2 (3)	0.005
White roots and tubers	5 (6)	1 (1)	ns	10 (13)	17 (22)	ns	10 (13)	14 (18)	ns
Organ meat	3 (4)	0 (0)	ns	1 (1)	0 (0)	ns	2 (3)	2 (3)	ns
Vitamin A-rich fruits	1 (1)	0 (0)	ns	2 (3)	10 (13)	0.017	1 (1)	12 (15)	0.001
Milk and milk products	1 (1)	0 (0)	ns	8 (10)	3 (4)	ns	9 (11)	3 (4)	ns

^1^ Intervention group had access to free model lunch provision on workdays through a canteen for six months (beginning of May until the end of October 2015) [3]. Evaluation in participants with complete data on workday dietary intake at all interviews. *p*-values from group comparisons using Fisher’s exact test; ^2^ *n* = 72 visited the canteen; ^3^ *n* = 71 visited the canteen; ns: not significant.

**Table 2:** Skipped meals and total 24-h food group intake on workdays at baseline and follow-up interviews in female Cambodian garment workers without (control) and with access to the free model lunch provision through a canteen (intervention). ^1^

	Baseline (End of April 2015)			At 2.5 Months (Middle of July 2015)			At 5 Months (Beginning of October 2015)		
	Control (*n* = 80)	Intervention (*n* = 78)		Control (*n* = 80)	Intervention (*n* = 78) ^2^		Control (*n* = 80)	Intervention (*n* = 78) ^3^	
Variables	*n* (%)	*n* (%)	*p*	*n* (%)	*n* (%)	*p*	*n* (%)	*n* (%)	*p*
Skipped meals									
Breakfast	9 (11)	7 (9)	ns	10 (13)	16 (21)	ns	12 (15)	11 (14)	ns
Lunch	0 (0)	0 (0)	-	0 (0)	0 (0)	-	0 (0)	0 (0)	-
Dinner	1 (1)	1 (1)	ns	0 (0)	2 (3)	ns	1 (1)	0 (0)	ns
Total 24-h food group intake									
Cereals	80 (100)	78 (100)	-	80 (100)	78 (100)	-	80 (100)	78 (100)	-
Spices, condiments and beverages	80 (100)	78 (100)	-	80 (100)	78 (100)	-	80 (100)	78 (100)	-
Oils and fats	75 (94)	66 (85)	ns	77 (96)	78 (100)	ns	75 (94)	78 (100)	ns
Flesh meats	73 (91)	68 (87)	ns	77 (96)	71 (91)	ns	75 (94)	72 (92)	ns
Other vegetables	69 (86)	58 (74)	ns	70 (88)	67 (86)	ns	69 (86)	70 (90)	ns
Fish and seafood	61 (76)	57 (73)	ns	61 (76)	59 (76)	ns	62 (78)	57 (73)	ns
Dark green leafy vegetables	60 (75)	57 (73)	ns	64 (80)	69 (89)	ns	49 (61)	68 (87)	<0.001
Sweets	53 (66)	53 (68)	ns	62 (78)	43 (55)	0.004	63 (79)	46 (59)	0.010
Other fruits	46 (58)	41 (53)	ns	55 (69)	71 (91)	0.001	58 (73)	66 (85)	ns
Vitamin A-rich vegetables and tubers	33 (41)	34 (44)	ns	39 (49)	43 (55)	ns	33 (41)	46 (62)	0.012
Legumes, nuts and seeds	27 (34)	26 (33)	ns	43 (54)	23 (30)	0.002	32 (40)	27 (35)	ns
Eggs	19 (24)	16 (21)	ns	30 (38)	35 (45)	ns	35 (44)	20 (26)	0.020
White roots and tubers	7 (9)	2 (3)	ns	13 (16)	18 (23)	ns	13 (16)	14 (18)	ns
Milk and milk products	4 (5)	4 (5)	ns	16 (20)	12 (15)	ns	19 (24)	18 (23)	ns
Organ meat	5 (6)	2 (3)	ns	7 (9)	7 (9)	ns	4 (5)	7 (9)	ns
Vitamin A-rich fruits	2 (3)	3 (4)	ns	2 (3)	11 (14)	0.009	2 (3)	14 (18)	0.001

^1^ Intervention group had access to free model lunch provision on workdays through a canteen for six months (beginning of May until the end of October 2015) [3]. Evaluation in participants with complete data on workday dietary intake at all interviews. *p*-values from group comparisons using Fisher’s exact test.; ^2^ *n* = 72 visited the canteen; ^3^ *n* = 71 visited the canteen; ns: not significant.
